# Application of 9.4T MRI in Wilson Disease Model TX Mice With Quantitative Susceptibility Mapping to Assess Copper Distribution

**DOI:** 10.3389/fnbeh.2020.00059

**Published:** 2020-04-22

**Authors:** Yongsheng Han, Jianjian Dong, Chenchen Xu, Rao Rao, Shan Shu, Guangda Li, Nan Cheng, Yun Wu, Hongyi Yang, Yongzhu Han, Kai Zhong

**Affiliations:** ^1^Hospital Affiliated to the Institute of Neurology Anhui University of TCM, Hefei, China; ^2^High Magnetic Field Laboratory, Hefei Institutes of Physical Science, Chinese Academy of Sciences (CAS), Hefei, China

**Keywords:** 9.4-Tesla magnetic resonance, penicillamine, quantitative susceptibility mapping, TX mouse, Wilson disease

## Abstract

In the current study, we used 9.4-tesla magnetic resonance imaging (9.4T MRI) and inductively coupled plasma mass spectrometry (ICP-MS) to investigate the distribution of copper in the brain samples of a murine model of Wilson’s disease (WD) following penicillamine (PCA) treatment. We also evaluated if the distribution of copper in the brain samples of mice was correlated with behavioral symptoms. Results from the behavioral experiments showed that 7 days of PCA treatment decreased the total distance traveled in the open field and the number of rearing and climbing instances among the toxic milk (TX) mice as compared with model group. We also observed that the open arm ratio in the elevated plus-maze (EPM) was reduced, escape latency in the Barnes maze test was increased, and avoidance in the open field was enhanced in TX mice following 14 days of PCA treatment as compared with those in untreated TX mice. We found that PCA treatment for 21–28 days improved the cognitive abilities, exploratory behavior, and movement behavior of TX mice. The PCA-treated mice also exhibited varying degrees of magnetic susceptibilities in the cortex, corpus striatum, hippocampus, and amygdaloid nucleus across the treatment period. Low copper concentrations were found in all of the analyzed brain regions of PCA-treated mice after 21–28 days as compared with the model group (*P* < 0.05). However, copper concentrations were increased in the primary motor cortex and cerebellum at 7 days post-PCA treatment as compared with those in the model group (*P* < 0.05). After 14 days of PCA treatment, the copper concentrations in the sensorimotor cortex, corpus striatum, hippocampus, and amygdaloid nucleus were higher than those detected without treatment. The results from a Pearson’s correlation analysis revealed that there was a significant (*P* < 0.05) correlation between copper concentrations and magnetic susceptibility in all of the brain regions that were analyzed. Therefore, our results indicate that copper concentration and magnetic susceptibility are associated with alterations in mood-related behavior, recognition memory, and movement behaviors in TX mice that are treated with PCA. The redistribution of copper in the TX mouse brain during PCA treatment may aggravate changes in behavioral performance.

## Introduction

Wilson’s disease (WD) is an autosomal recessive copper metabolism disorder that is characterized by the dysfunction of copper metabolism. Specifically, the ATP7b gene mutation decreases the binding of copper to ceruloplasmin (CP) and reduces bile excretion (Członkowska et al., 2018). This condition results in the deposition of large amounts of copper in vital organs, such as the liver, brain, and kidney. Clinically, WD manifests as acute and chronic liver injury, neurological/psychiatric symptoms, and renal impairment symptoms (Ala et al., 2007; Capone and Azzam, 2018). Additionally, copper chelators, such as penicillamine (PCA), zinc salts, or combinations of these agents, are commonly administered to patients with WD to remove excess copper from the organs (Aggarwal and Bhatt, 2014). However, PCA may induce serious adverse reactions in patients with WD and even worsen their neurological symptoms (Walshe and Yealland, 1993). Although the mechanisms underlying the neurological aggravation following PCA treatment have not been verified, prior research has suggested that the free flow of copper into circulating blood and other organs following the rapid transfer of copper from organs after the initiation of chelating treatments may play a role in this phenomenon (Stuerenburg, 2000; Litwin et al., 2019).

Magnetic resonance post-processing techniques, such as quantitative magnetic susceptibility imaging (QSM), allow for the detection of copper in brain tissue and the investigation of the distribution of residual copper. Additionally, studies have reported that QSM provides sensitive evaluations of paramagnetic metallic compounds and diamagnetic substances, such as calcifications, in the brain and effectively detects copper accumulation in the brains of patients with WD (Fritzsch et al., 2014).

The present study used QSM and inductively coupled plasma mass spectrometry (ICP-MS) to quantify in the concentrations of copper in the brain of a PCA-treated murine model of WD and investigate the effect of PCA treatment on copper-induced cognitive decline, locomotor behavior impairment, and anxiety-like behaviors in these mice.

## Materials and Methods

### Animals and Ethical Statements

The C3HeB/FeJ-Atp7b^tx-J/J^ (TX) mouse is a naturally occurring genetic and phenotypic model of WD that is derived from the background strain C3HeB/FeJ (DL) mouse. The TX mouse model has a G712D missense mutation in the ATP7b copper transporter gene that results in a phenotype that is similar to WD (Coronado et al., 2001; Roberts et al., 2008). In the current study, mice (age, 4–5 months; weight, 25–30 g) were obtained from the Jackson Laboratory in the United States. All of the animals were housed in standard laboratory cages (3–5 mice per cage) under standard laboratory conditions (12 h light/dark cycle, 20–22°C, 50%–60% humidity) before the experiments. The mice had *ad libitum* access to water and food, and this study was approved by the Anhui University of Chinese Medicine Animal Ethics Committees and was conducted following the Chinese Council on Animal Care Guidelines.

### Study Design

PCA was dissolved in deionized water and orally administered to TX mice at a dose of 25 mg/kg/days for 7, 14, 21, and 28 days respectively. It means that 1 group received PCA for 7 consecutive days, another group for 14 days, the third group for 21 days, and the final group for 28 days. The dose of PCA was selected by converting the human dose into a mouse dose. The mice were divided into six groups (nine mice/group). The 7th, 14th, 21st, and the 28th-day group consisted of TX mice that were treated with PCA, the control group consisted of DL mice that received oral administrations of deionized water, and the model group consisted of TX mice that received oral administrations of deionized water. The cognitive and activity functions in each mouse were assessed on day 7, 14, 21, and 28.

### Elevated Plus-Maze (EPM)

The elevated plus-maze (EPM) apparatus consists of two closed arms (30 cm × 15 cm × 5 cm) and two open arms (30 cm × 5 cm) that extend from a central platform (5 × 5 cm) at an elevation of 75 cm. Twelve hours after the administration of PCA on the 7th, 14th, 21st, and 28th days, each mouse was placed on the edge of an open arm facing the central platform, and the transfer latency was tested for 5 min. All experimental procedures were tested and analyzed using the Ethovision XT monitoring and analysis system (Noldus, Wageningen, Netherlands). The percentage of time that the mice remained in the open arm (the total time spent in the open arm) and the percentages in the open arms (the total number of times entering the open arms) were used as indicators of anxiety.

### Open Field Test (OFT)

The open field test (OFT) equipment consisted of a white, square field (50 × 50) that was divided into smaller squares that were 5 × 5 cm in area. The mice were gently placed in the center of the test room, recorded for 5 min, and monitored using an automatic video tracking system. The Noldus animal behavior analysis program was used to automatically analyze and generate a digital image of the path taken by the mice within 5 min. After each test, the arena was cleaned with a 70% ethanol solution.

### Barnes Maze

The Barnes maze was used to test the spatial learning and memory abilities of the mice by subjecting them to a habitual experiment. On the first day, the experimenter immediately guided the mouse to a shelter after placing it onto the platform. This was followed by a 4-day space acquisition phase whereby the mouse was allowed to explore the apparatus freely for 3 min each day; if the mouse did not arrive at the shelter during the 3 min of exploration, it was manually guided there by the experimenter. A probe test was performed on day 5. During the probe test, the animals were able to explore the maze while all of the holes, including the hole that led to the shelter, were closed. The experimenter cleaned the platform and escape box with 70% ethanol after each experiment to eliminate odor. The time taken for each mouse to find the target box in each trial (latency) was recorded with the Noldus Maze Video Tracking System.

### MR Image Acquisition

**Figure 1:**
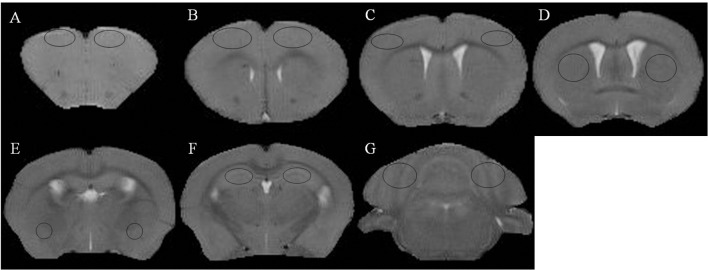
T2 weighted images and regions of interest.** (A)** Prefrontal cortex. **(B)** Primary motor cortex. **(C)** Sensorimotor cortex. **(D)** Corpus striatum. **(E)** Amygdaloid nucleus. **(F)** Hippocampus. **(G)** Cerebellum.

The mice were anesthetized with 3.5% isoflurane mixed with oxygen, placed on their back in the animal bed, and kept warm with a water bath pad. During the scanning period, the mice were anesthetized with 0.8%–1% isoflurane, and a breath monitoring system was used to observe the vital signs of the mice, and maintain their respiratory rate at 30 ± 5 times/min. Images were scanned with the Agilent Technologies 9.4T/400PS animal scanner (Agilent Technologies, Santa Clara, CA, USA), which features a caliber of 40 cm. The gradient coil had a diameter of 26 cm. We used a self-made, high-sensitivity, small-sized, orthogonal, high-pass RF coil. The T2 weighted images (Figure 1) and phase images were obtained by rapid acquisition with a relaxation enhancement (RARE) sequence. The related parameters were as follows: repetition time (TR) = 5,000 ms, echo spacing (ESP) = 11.1 ms, field of vision (FOV) = 16 mm × 16 mm, bandwidth = 40 kHz, echo train length (ETL) = 8, k-zero = 3, effective echo time (TE) = 33, averages = 6, slices = 20, thickness = 0.5 mm, gap = 0 mm, matrix = 192 × 192; The protocol parameters for the multi-echo spoiled gradient echo (GRE) sequence were as follows: TR = 800 ms, TE = 3.01 ms, TE_2_ = 4.39 ms, echos = 5, flip angle (FA) = 35°, FOV = 16 mm × 16 mm, bandwidth = 78 kHz, averages = 3, slices = 20, thickness = 0.5 mm, gap = 0 mm, and matrix = 192 × 192. The typical spectra line widths for water resonance after map shimming were approximately 20 Hz.

**Figure 2:**
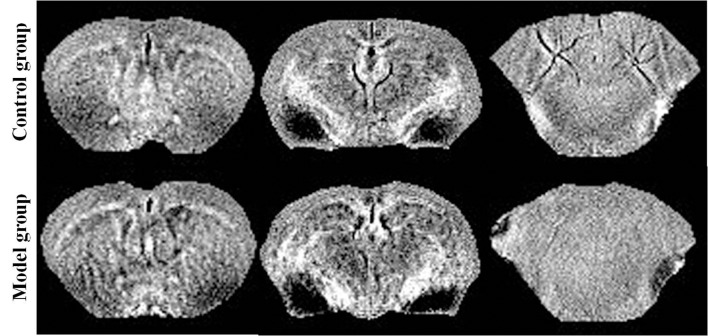
Quantitative susceptibility mapping at the same slice of the control/model group.

Reconstruction of the QSM image from the multi-echo GRE image consisted of phase unwrapping, the removal of the background phase with the projection onto dipole fields (PDF) approach, and the reconstruction of the magnetic susceptibility map using morphology-enabled dipole inversion (MEDI). The region of interest (ROI; Figure 1) analyses of the cortex, corpus striatum, hippocampus, amygdaloid nucleus, and cerebellum were performed on each side of the QSM images (Figure 2). The ROI was manually drawn by a neuroradiologist with ImageJ software on the layer at which the ROI was most visible. Each ROI was divided according to its anatomical boundary. Since the magnetic susceptibility of the cerebrospinal fluid is not anisotropic relative to that of white matter, reference measurements were only obtained in ROIs that contained cerebrospinal fluid.

### Quantitative Mineral Analysis

Brain tissue was digested in a microwave digestion system with 3 ml of nitric acid at 130°C. The digested sample was cooled at room temperature for 4 h. The extract was diluted with 3 ml of deionized water. Copper content was determined with ICP-MS (Perkin Elmer 350D). All of the reagents used were of analytical reagent grades (Perkin Elmer, Germany), and the accuracy and precision of the analytical methods were verified with standard controls (Perkin Elmer, Germany).

### Statistical Methods

Statistical analyses were performed using Graph Pad Prism software version 5, and all of the data are expressed as the mean ± standard error of the mean (SEM). An analysis of variance (ANOVA) and multiple comparisons were used to compare differences in the behavioral analyses, the concentrations of copper and magnetic susceptibilities between the six groups. Pearson’s correlation analysis was used to assess the correlation between copper concentration and magnetic susceptibility, copper concentration and behavior, and magnetic susceptibility and behavior. For all analyses, *P* < 0.001 indicated highly significant differences ***P < 0.01 indicated moderately significant differences **P < 0.05 indicated significant differences *P > 0.05 indicated no significant difference.

## Results

### Effects of PCA on Spatial Learning and Memory Abilities of the TX Mouse

**Figure 3:**
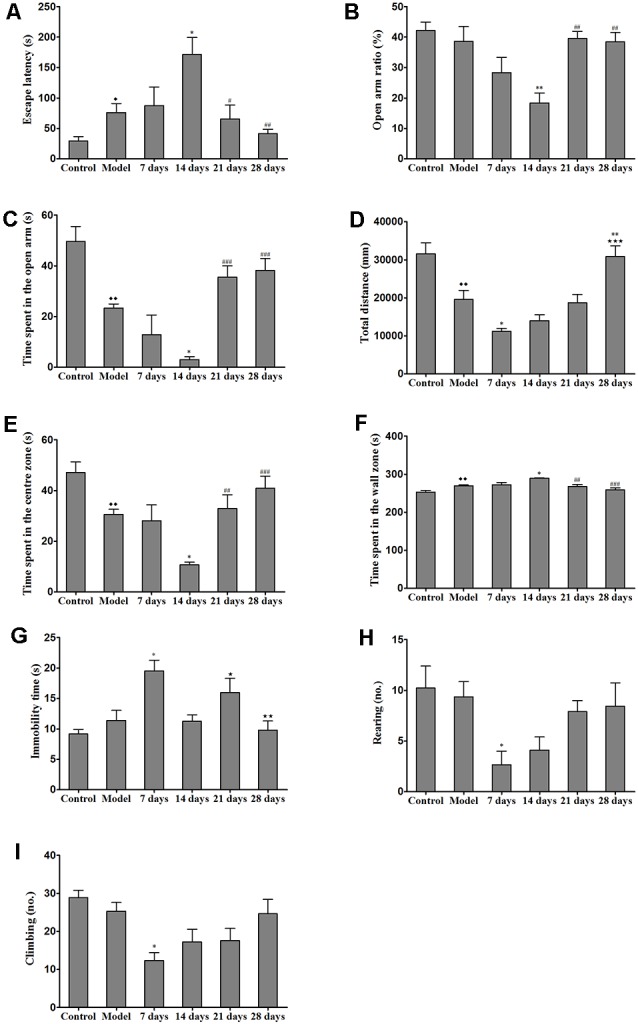
Effects of penicillamine (PCA) on behavior in toxic milk (TX) mice. **(A)** The escape latency of the model group was increased as compared with that of the wild-type control group. The escape latency of the TX mice after 14 days of PCA treatment was prolonged. The escape latencies of TX mice were decreased on the 21st and 28th days of PCA treatment as compared with those of TX mice on the 14th day of PCA treatment. **(B)** The open arm ratio of the TX mice decreased after 14 days of PCA treatment. On the 21st and 28th days of PCA treatment, the open arm ratio was significantly increased as compared with that on the 14th day of PCA treatment. **(C)** The amount of time spent in the open arms in the model group was significantly decreased as compared with that in the control group. The amount of time spent in the open arms was significantly decreased on the 14th day of PCA administration and increased on the 21st and 28th days of PCA treatment in the TX mice as compared with that in the model group. **(D)** The total distance traveled by the TX mice was significantly decreased as compared with the total distance traveled by the control group. The total distance traveled by the TX mice decreased after 7 days of PCA treatment. The total distance traveled was significantly increased on the 28th day of PCA treatment as compared with that on the 7th day of PCA treatment. **(E)** The amount of time spent in the center zone by the model group was significantly decreased as compared with the amount of time spent in the center zone by the control group. The amount of time spent in the center zone decreased on the 14th day of PCA treatment, and significantly increased on the 21st and 28th days of PCA treatment. **(F)** Mice in the model group spent significantly more time in the wall zone than did mice in the control group. The amount of time spent in the wall zone increased on the 14th day of PCA treatment, and decreased on the 21st and 28th days of PCA treatment. **(G)** The immobility time of TX mice was significantly increased on the 7th day of PCA treatment and significantly decreased on the 14th and 28th days of PCA treatment as compared with the immobility time of the model group. **(H)** The number of rearing instances in TX mice was significantly decreased on the 7th day of PCA treatment as compared with that in the model group. **(I)** On the 7th day of PCA treatment, the number of climbing instances was significantly decreased as compared with that in the model group. Data are presented as the mean ± standard error of the mean (SEM); one-way ANOVA and Bonferroni’s *post hoc* test, ^⧫^*P* < 0.05, ^⧫⧫^*P* < 0.01 vs. the corresponding control group; *P < 0.05, **P < 0.01 vs. the corresponding model group; ^⋆^*P* < 0.05, ^⋆⋆^*P* < 0.01, ***P < 0.001 vs. the corresponding 7th-day group; ^#^*P* < 0.05, ^##^*P* < 0.01, ^###^*P* < 0.001 vs. the corresponding 14th-day group.

After the mice were habituated to the Barnes maze, we evaluated spatial learning and memory abilities, which were indicated by the latencies to reach the target hole and enter the escape hole, in all of the groups (e.g., the control group, model group and PCA-treatment groups) during the 28 days of testing. We found that the escape latency of the PCA-treated mice was significantly longer than that of the TX mice on the 14th day of testing (*P* < 0.05). On the 21st and 28th days, PCA-treated mice showed enhanced spatial learning and memory abilities in the Barnes maze task as compared with those of untreated TX mice (Figure 3A).

### Effects of PCA on Movement Behaviors, Anxiety-Related Behaviors, and Locomotor Activity

We assessed the effects of PCA on activity and anxiety-related behavior in mice were assessed with EPM. Results from the EPM revealed that 14 days of PCA treatment caused a significant decrease (*P* < 0.01) in the open-arm ratio and time spent in the open arms in the TX mice as compared with those in untreated TX mice (Figure 3). However, anxiety-related behavior was improved on the 21st and 28th days of PCA administration (*P* < 0.01). To test whether the learning impairments exhibited by the PCA-treated mice were attributable to the lack of natural anxiety-related behaviors, we evaluated the characteristics of anxiety-related behaviors using the OFT after PCA treatment. Our results revealed that the total distance traveled by the TX mice was decreased after PCA treatment for 7 days (*P* < 0.05) and increased after 28 days (*P* < 0.001; Figure 3D). Additionally, the PCA-treated mice spent significantly less time in the center zone and more time in the wall zone than did the untreated TX mice on the 14th day (Figures 3E,F). Our data also showed that PCA treatment for 21 days and 28 days significantly increased the time spent in the center zone and decreased the time spent in the wall zone by the TX mice (*P* < 0.01). Meanwhile, the instances of rearing and climbing behaviors were reduced in TX mice after 7 days of PCA treatment as compared with those in the model group (Figures 3H,I).

### Total Copper Concentration in the TX Mouse Brain Samples

**Figure 4:**
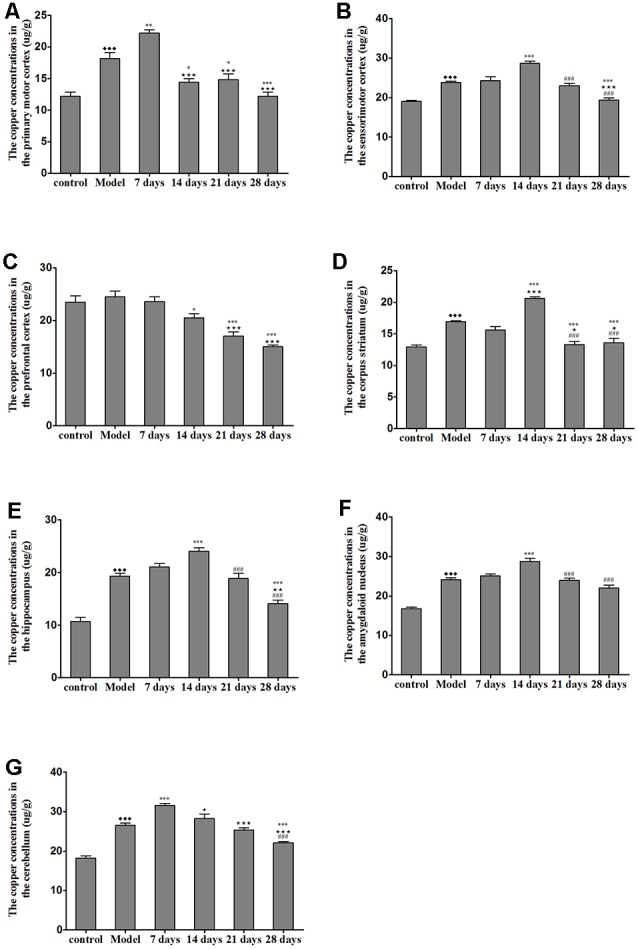
Total copper concentration in TX mouse brain samples after PCA treatment.** (A**,**B)** The copper concentrations in the primary motor cortex were significantly increased in TX mice after 7 days as compared with those in mice in the model group. Additionally, the copper concentrations in the sensorimotor cortex were significantly increased in TX mice after 14 days of treatment with PCA and decreased on the 28th day of treatment as compared with those in the model group. **(C)** The copper concentrations in the prefrontal cortex of TX mice decreased with PCA administration. **(D–F)** The copper concentrations in the corpus striatum, hippocampus, and amygdaloid nucleus of TX mice increased on the 14th day of treatment and decreased on the 21st and 28th days of treatment. **(G)** The copper concentrations in the cerebellum of TX mice increased on the 7th day of treatment and decreased on the 21st and 28th days of treatment. Data are presented as the mean ± SEM; *n* = 9 animals/group; one-way ANOVA, and Bonferroni’s *post hoc* test, ^⧫⧫⧫^*P* < 0.001 vs. the corresponding control group; *P < 0.05, **P < 0.01, ***P < 0.001 vs. the corresponding model group; ^⋆^*P* < 0.05, ^⋆⋆^*P* < 0.01, ^⋆⋆⋆^*P* < 0.001 vs. the corresponding 7th day group; ^###^*P* < 0.001 vs. the corresponding 14th day group.

The concentration of copper in all of the regions measured was significantly increased in the untreated TX mice as compared with the healthy controls. Additionally, the concentrations of copper in the sensorimotor cortex, amygdaloid nucleus, corpus striatum, and hippocampus were significantly elevated on the 14th day of PCA administration as compared with those in the untreated TX mice (*P* < 0.05); however, the copper concentrations in the PCA-treated mice were decreased on the 21st and 28th days of treatment as compared with those in untreated TX mice. The copper concentrations in the primary motor cortex and cerebellum of TX mice were significantly increased on the 7th day of PCA treatment (*P* < 0.05) and gradually decreased on the 21st and 28th days of treatment (Figure 4).

### Effects of PCA on Magnetic Susceptibility Using 9.4T MRI

**Figure 5:**
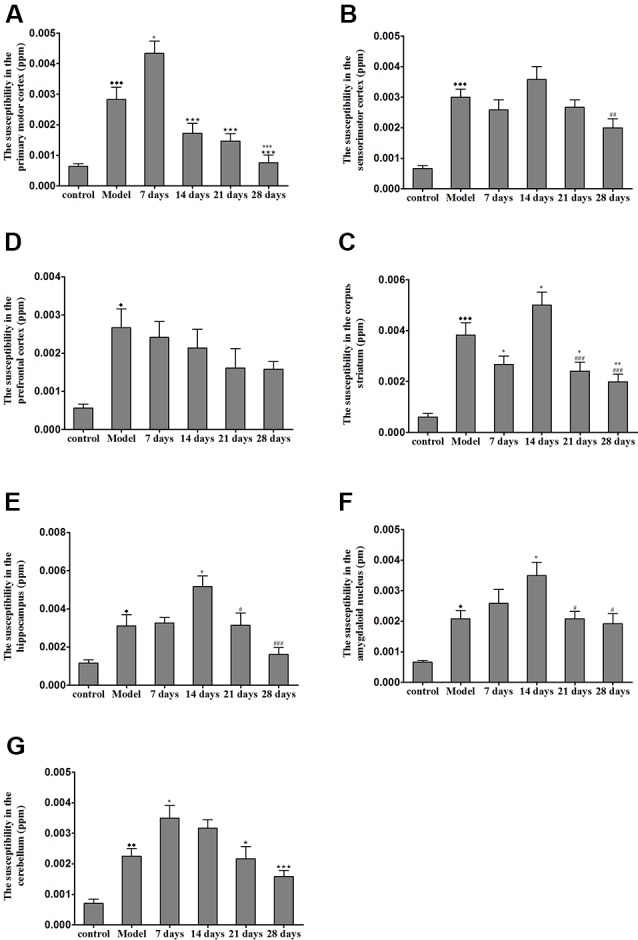
Susceptibility values in various regions of TX mice brain after PCA treatment.** (A)** The magnetic susceptibility in the primary motor cortex of the TX mice was increased as compared with that in the primary motor cortex of mice in the control group. The magnetic susceptibility increased in the primary motor cortex after 7 days of treatment with PCA and decreased on 14, 21, 28 days of PCA treatment. **(B)** The magnetic susceptibility in the sensorimotor cortex of the TX mice was significantly increased as compared with that in the sensorimotor cortex of mice in the control group. **(C)** The magnetic susceptibility of the TX mice in the prefrontal cortex was increased as compared with that of mice in the control group. **(D–F)** The magnetic susceptibility of the TX mice in the corpus striatum, hippocampus, and amygdaloid nucleus, respectively, was increased as compared with that of mice in the control group. The magnetic susceptibility in the corpus striatum, hippocampus, and amygdaloid nucleus of TX mice increased on the 14th day and decreased on the 21st and 28th days. **(G)** The magnetic susceptibility in the cerebellum of TX mice was increased as compared with that in the cerebellum of mice in the control group. The magnetic susceptibility in the cerebellum of TX mice increased on the 7th day and decreased on the 21st and 28th days. Data are presented as the mean ± SEM; *n* = 6 animals/group; one-way ANOVA, and Bonferroni’s *post hoc* test, ^⧫^*P* < 0.05, ^⧫⧫^*P* < 0.01, ^⧫⧫⧫^*P* < 0.001 vs. the corresponding control group; *P < 0.05, **P < 0.01, ***P < 0.001 vs. the corresponding model group; ^⋆^*P* < 0.05, ^⋆⋆⋆^*P* < 0.001 vs. the corresponding 7th day group; ^#^*P* < 0.05, ^##^*P* < 0.01, ^###^*P* < 0.001 vs. the corresponding 14th day group.

The brain magnetic susceptibility of TX mice was significantly higher than that of the control mice. Additionally, we found that 7 days of PCA treatment increased the magnetic susceptibility in the primary motor cortex and cerebellum (*P* < 0.05); however, the magnetic susceptibility in the primary motor cortex and cerebellum gradually decreased on the 21st and 28th days of PCA treatment. Fourteen days of PCA treatment increased the susceptibility in the amygdaloid nucleus, hippocampus, and corpus striatum (*P* < 0.05), then decreased after 28 days (*P* < 0.01; Figure 5).

### Correlation Between Copper Concentration and Magnetic Susceptibility

**Figure 6:**
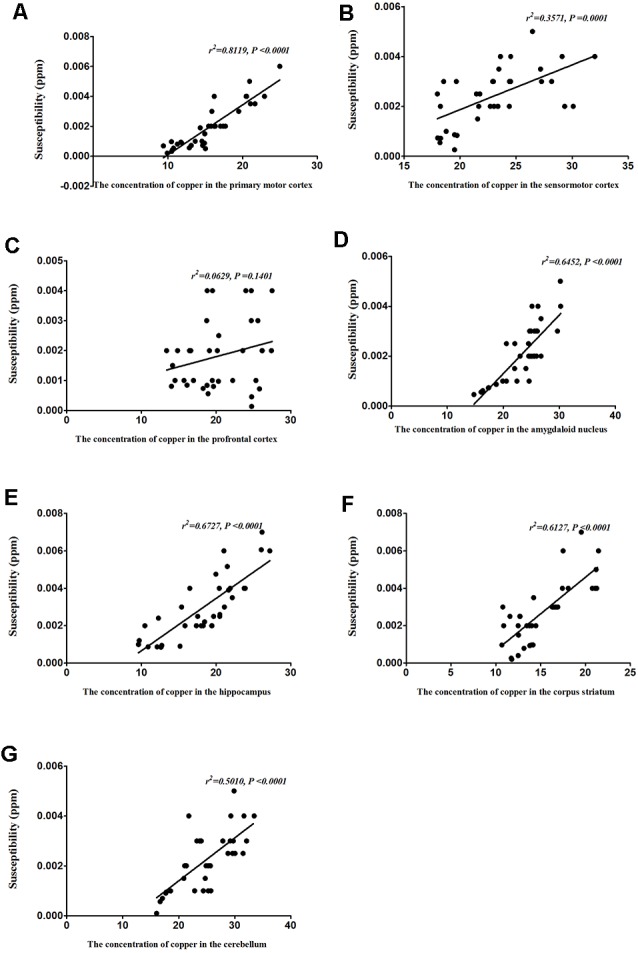
Correlation between copper concentrations and magnetic susceptibility.** (A)** Correlation between magnetic susceptibility and copper concentrations in the primary motor cortex. **(B)** Correlation between magnetic susceptibility and copper concentrations in the sensorimotor cortex. **(C)** Correlation between magnetic susceptibility and copper concentrations in the prefrontal cortex. **(D)** Correlation between magnetic susceptibility and copper concentrations in the amygdaloid nucleus. **(E)** Correlation between magnetic susceptibility and copper concentrations in the hippocampus. **(F)** Correlation between magnetic susceptibility and copper concentrations in the corpus striatum. **(G)** Correlation between magnetic susceptibility and copper concentrations in the cerebellum.

To identify the contribution of copper to the magnetic susceptibility of the brain, we assessed the correlations between magnetic susceptibility and copper concentrations in all TX mice groups. Results from our Pearson’s correlation analysis showed that there was a positive correlation between magnetic susceptibility and copper concentrations in the primary motor cortex (*r*^2^ = 0.8119, *P* < 0.0001), sensorimotor cortex (*r*^2^ = 0.3571, *P* = 0.0001), amygdaloid nucleus (*r*^2^ = 0.6452, *P* < 0.0001), hippocampus (*r*^2^ = 0.6727, *P* < 0.0001), corpus striatum (*r*^2^ = 0.6127, *P* < 0.0001), and cerebellum (*r*^2^ = 0.5010, *P* < 0.0001) of DL/TX mice (Figure 6).

### Correlation Between Behavioral Alterations and Copper Concentrations

**Figure 7:**
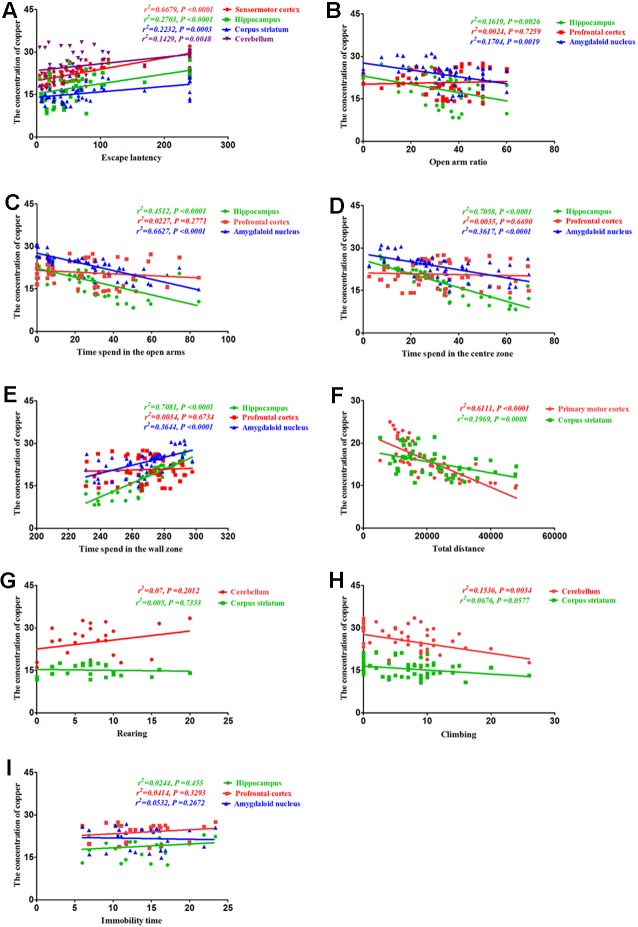
Correlation between behavior and copper concentrations.** (A)** Correlation between escape latency and copper concentrations. Escape latencies were significantly correlated with copper concentrations in the sensorimotor cortex, hippocampus, corpus striatum, and cerebellum. **(B)** Correlation between the open arm ratio and copper concentrations. The open arm ratio was significantly correlated with copper concentrations in the hippocampus and amygdaloid nucleus, but no significant correlations were observed between the open arm ratio and copper concentrations in the prefrontal cortex. **(C)** Correlation between the amount of time spent in the open arms of the elevated plus-maze (EPM) and copper concentrations. The amount of time spent in the open arms was significantly correlated with copper concentrations in the hippocampus and amygdaloid nucleus, but no significant correlations were observed between the amount of time spent in the open arms and copper concentrations in the prefrontal cortex. **(D)** Correlation between the amount of time spent in the center zone and copper concentrations. The amount of time spent in the center zone was significantly correlated with copper concentrations in the hippocampus and amygdaloid nucleus, but no significant correlations were observed between the amount of time spent in the center zone and copper concentrations in the prefrontal cortex. **(E)** Correlation between the amount of time spent in the wall zone and copper concentrations. The amount of time spent in the wall zone was significantly correlated with copper concentrations in the hippocampus and amygdaloid nucleus, but no significant correlations were observed between the amount of time spent in the wall zone and copper concentrations in the prefrontal cortex. **(F)** Correlation between the total distance traveled and copper concentrations. The total distance traveled was significantly correlated with copper concentrations in the primary motor cortex and corpus striatum. **(G)** Correlation between rearing and copper concentrations. There were no significant correlations between rearing and copper concentrations in the cerebellum and corpus striatum. **(H)** Correlation between climbing and copper concentrations. Climbing was significantly correlated with copper concentrations in the cerebellum, but no significant correlations were observed between climbing and copper concentrations in the corpus striatum. **(I)** Correlation between immobility time and copper concentrations. No obvious correlation was found between immobility time and copper concentrations in the hippocampus, prefrontal cortex, and amygdaloid nucleus.

To determine whether the altered behavior of PCA-treated mice was associated with copper distribution, we assessed the correlations between the behavioral alterations induced by PCA treatment and copper concentrations in various brain regions. Our results demonstrated that the escape latencies were significantly correlated with copper concentrations in the sensorimotor cortex (*r*^2^ = 0.6679, *P* < 0.0001), hippocampus (*r*^2^ = 0.2703, *P* < 0.0001), corpus striatum (*r*^2^ = 0.2232, *P* = 0.0003), and cerebellum (*r*^2^ = 0.1429, *P* = 0.0048; Figure 7A). Additionally, we observed a significant correlation between the open arm ratio and copper concentrations in the hippocampus (*r*^2^ = 0.1619, *P* = 0.0026), and amygdaloid nucleus (*r*^2^ = 0.1704, *P* = 0.0019; Figure 7B). The amount of time spent in the open arms was also significantly correlated with copper concentrations in the hippocampus (*r*^2^ = 0.7081, *P* < 0.0001) and amygdaloid nucleus (*r*^2^ = 0.3644, *P* < 0.0001); however, no significant correlations were observed between the amount of time spent in the open arms and copper concentrations in the prefrontal cortex (*r*^2^ = 0.0227, *P* = 0.2771; Figure 7C). Results from the Pearson’s correlation analysis showed that there was a negative correlation between the amount of time spent in the center zone and copper concentrations in the hippocampus (*r*^2^ = 0.7058, *P* < 0.0001) and amygdaloid nucleus (*r*^2^ = 0.3617, *P* < 0.0001), and time spent in the center zone (Figure 7D); however, there was a positive correlation between the amount of time spent in the wall zone and copper concentrations in the hippocampus and amygdaloid nucleus (*r*^2^ = 0.7081, *P* < 0.0001; *r*^2^ = 0.3644, *P* < 0.0001; Figure 7E). The total distance traveled was significantly correlated with copper concentration in the primary motor cortex (*r*^2^ = 0.6574, *P* < 0.0001; Figure 7F) and corpus striatum (*r*^2^ = 0.1969, *P* = 0.0008; Figure 7F), but no significant correlations were observed between rearing and copper concentrations in the cerebellum (*r*^2^ = 0.07, *P* = 0.2012) and corpus striatum (*r*^2^ = 0.005, *P* = 0.7353; Figure 7G). The copper concentration in the cerebellum of PCA-treated mice was negatively associated with climbing (*r*^2^ = 0.1536, *P* = 0.0034; Figure 7H), and no obvious correlation was found between immobility time and copper concentrations in the hippocampus (*r*^2^ = 0.0245, *P* = 0.455), prefrontal cortex (*r*^2^ = 0.0391, *P* = 0.3433) and amygdaloid nucleus (*r*^2^ = 0.0025, *P* = 0.8139; Figure 7I).

### Correlation Between Behavioral Alterations and Magnetic Susceptibility

**Figure 8:**
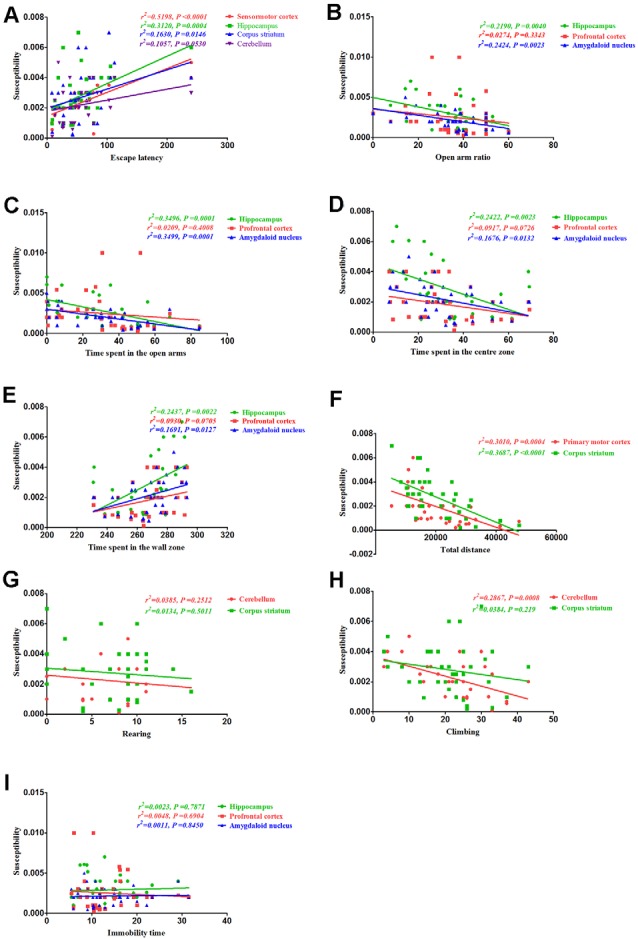
Correlation between behavior and magnetic susceptibility.** (A)** Correlation between escape latency and magnetic susceptibility. The escape latencies were significantly correlated with magnetic susceptibility in the sensorimotor cortex, hippocampus, and corpus striatum, but no significant correlations were observed between the escape latencies and magnetic susceptibility in the cerebellum. **(B)** Correlation between the open arm ratio and magnetic susceptibility. The open arm ratio was significantly correlated with magnetic susceptibility in the hippocampus and amygdaloid nucleus, but no significant correlations were observed between the open arm ratio and magnetic susceptibility in the prefrontal cortex. **(C)** Correlation between the amount of time spent in the open arms of the EPM and magnetic susceptibility. The amount of time spent in the open arms was significantly correlated with magnetic susceptibility in the hippocampus and amygdaloid nucleus, but no significant correlations were observed between the amount of time spent in the open arms and magnetic susceptibility in the prefrontal cortex. **(D)** Correlation between the amount of time spent in the center zone and magnetic susceptibility. The amount of time spent in the center zone was significantly correlated with magnetic susceptibility in the hippocampus and amygdaloid nucleus, but no significant correlations were observed between the amount of time spent in the center zone and magnetic susceptibility in the prefrontal cortex. **(E)** Correlation between the amount of time spent in the wall zone and magnetic susceptibility. The amount of time spent in the wall zone was significantly correlated with magnetic susceptibility in the hippocampus and amygdaloid nucleus, but no significant correlations were observed between the amount of time spent in the wall zone and magnetic susceptibility in the prefrontal cortex. **(F)** Correlation between the total distance traveled and magnetic susceptibility. The total distance traveled was significantly correlated with the magnetic susceptibility in the primary motor cortex and corpus striatum. **(G)** Correlation between rearing and magnetic susceptibility. There were no significant correlations were rearing and magnetic susceptibility in the cerebellum and corpus striatum. **(H)** Correlation between climbing and magnetic susceptibility. Climbing was significantly correlated with magnetic susceptibility in the cerebellum, but no significant correlations were observed between climbing and magnetic susceptibility in the corpus striatum. **(I)** Correlation between immobility time and magnetic susceptibility. There were no obvious correlations between immobility time and magnetic susceptibility in the hippocampus, prefrontal cortex, and amygdaloid nucleus.

Finally, we assessed the correlation between behavioral alterations induced by PCA and magnetic susceptibility. Our results revealed that the escape latencies were significantly correlated with magnetic susceptibility in the sensorimotor cortex (*r*^2^ = 0.5198, *P* < 0.0001), hippocampus (*r*^2^ = 0.312, *P* = 0.0004) and corpus striatum (*r*^2^ = 0.163, *P* = 0.0146), but no significant correlations were observed between escape latencies and magnetic susceptibility in the cerebellum (*r*^2^ = 0.1057, *P* = 0.053; Figure 8A). Additionally, the open arm ratio was significantly correlated with the copper concentration in the hippocampus (*r*^2^ = 0.219, *P* = 0.004) and amygdaloid nucleus (*r*^2^ = 0.2424, *P* = 0.0023; Figure 8B). Further, there was a significant correlation between the amount of time spent in the open arms and magnetic susceptibility in the hippocampus (*r*^2^ = 0.3496, *P* = 0.0001) and amygdaloid nucleus (*r*^2^ = 0.3499, *P* = 0.0001; Figure 8C). Results from the Pearson’s correlation analysis showed that there was a negative correlation between the amount of time spent in the center zone and magnetic susceptibility in the hippocampus (*r*^2^ = 0.2422, *P* = 0.0023) and amygdaloid nucleus (*r*^2^ = 0.1676, *P* = 0.0132; Figure 8D); however, there was a positive correlation between the amount of time spent in the wall zone and magnetic susceptibility in the hippocampus and amygdaloid nucleus (Figure 8E). The total distance traveled was significantly correlated with the magnetic susceptibility in the primary motor cortex (*r*^2^ = 0.3101, *P* = 0.0004) and corpus striatum (*r*^2^ = 0.3687, *P* < 0.0001; Figure 8F). Although magnetic susceptibility in the cerebellum of PCA-treated mice was negatively associated with climbing (*r*^2^ = 0.2867, *P* < 0.0001; Figure 8H), rearing was not significantly correlated with magnetic susceptibility (*r*^2^ = 0.0385, *P* = 0.2512; Figure 8G). There were no significant correlations between immobility time and magnetic susceptibility in the hippocampus (*r*^2^ = 0.0023, *P* = 0.7871), prefrontal cortex (*r*^2^ = 0.0047, *P* = 0.6904) and amygdaloid nucleus (*r*^2^ = 0.0011, *P* = 0.845; Figure 8I).

## Discussion

This study examined whether PCA treatment exacerbated copper-induced short-term memory deficits, anxiety-like behaviors, and movement behaviors in the TX mouse model, which is a model that is commonly used to explore the deleterious effects of PCA administration on the neurological symptoms of WD. We found that PCA treatment worsened spatial learning and memory, anxiety-like behaviors, and locomotor activity in the early stages; however, the extent of these effects was reduced after prolonged treatment.

PCA is a non-specific metal chelating agent that is commonly used to treat WD by gradually increasing the prescribed dose from a relatively low baseline dose to a therapeutically effective dose (Joyce, 1990). While the modality of PCA treatment may aggravate neurological symptoms, continued treatment or changes in the prescribed dose may improve the condition (Kathawala and Hirschfield, 2017). For example, Walshe (Walshe and Yealland, 1993) found that PCA was effective in 97 out of 137 patients with WD who presented with neurological symptoms; however, 24 patients had a poor response to PCA treatment, and 20 patients died. Moreover, neurological symptoms worsened in 30 patients during the initial treatment period before improvement. Additionally, Brewer et al. (1987) found evidence suggesting that the PCA-induced worsening of WD is at least 50% irreversible.

It is worth noting that the aggravation of neurological symptoms that are observed with PCA treatment is also observed with other therapeutic drugs, such as trientine (Boga et al., 2015; Li et al., 2016). Although researchers discovered that PCA improves language dysfunction, tremor, muscle rigidity, bradykinesia, and salivation resultant of WD, they also revealed that PCA is useless or even detrimental to various symptoms, including dysphagia, sputum torsion, and dance-like involuntary movements (Schilsky, 2017; Mohr and Weiss, 2019).

The present study further demonstrated that TX mice showed significant spatial learning and memory impairment in the Barnes maze test and the EPM test after 14 days of PCA treatment. Additionally, results from the OFT revealed that the TX mice exhibited anxiety-like behavior and locomotor activity impairment after 7 days of PCA treatment. Further, these behavioral phenotypes, which were associated with copper accumulation, worsened with PCA treatment during the early stage of the disease.

Although the causes and mechanisms underlying the PCA-induced exacerbation of symptoms have not been fully elucidated, multiple hypotheses that explain this phenomenon have been accepted. For example, one hypothesis suggests that copper in the body will be redistributed after treatment with PCA copper-displacement, and the subsequent deposition of large amounts of copper in brain tissue can cause symptoms that are similar to or more severe than the original neurological symptoms (Walshe and Yealland, 1993). According to another hypothesis, copper can continuously stimulate neural tissue during its induced transfer, which may cause blood-brain barrier dysfunction in patients with WD. Meanwhile, copper can bind to drugs in the bloodstream, mediate their passage through the damaged blood-brain barrier, and ultimately aggravate brain-tissue damage (Stuerenburg, 2000). Another hypothesis states that since the deposition of copper in nerve tissue may not be reversed in some patients with WD (even with the use of copper-loading drugs, such as PCA) the neurological symptoms of these patients are irreversible, and PCA may even accelerate them (Appenzeller-Herzog et al., 2019).

We further analyzed whether the harmful effects of PCA treatment on behavior were accompanied by the redistribution of copper deposition in the brain. Our data indicated that copper concentrations were higher in the cortex, hippocampus, corpus striatum, amygdaloid nucleus, and cerebellum after 7 and 14 days of treatment with PCA. Our data further also demonstrated that the escape latency was significantly correlated with copper concentrations in the sensorimotor cortex hippocampus, and corpus striatum. These results suggest that the increased cortical, striatal and hippocampal copper concentrations in TX mice following PCA treatment may contribute to the observed memory deficits. Further, our findings revealed that copper concentrations and magnetic susceptibilities in the hippocampi and amygdaloid nucleus of TX mice were positively correlated with the amount of time spent in the wall zone; however, copper concentrations and magnetic susceptibilities in the hippocampi and amygdaloid nucleus of TX mice were negatively correlated with the amount of time spent in the center zone. Also, the amount of time spent in the open arms was correlated with copper concentrations and magnetic susceptibility in the hippocampi and amygdaloid nucleus. Hence, our data suggest that the increased copper levels in the hippocampus and amygdaloid nucleus may contribute to anxiety-like behavior in TX mice after 14 days of PCA treatment. Movement and locomotor impairments in TX mice were also concurrently observed with the rises in the susceptibility levels in the primary motor cortex and cerebellum after 7 days of PCA treatment. Therefore, our data demonstrated that the increased copper levels in the cortex and cerebellum may contribute to impairments in movement behavior and locomotor activity.

QSM is a post-processing technology that uses a high pass filter phase image to distinguish between the influences of paramagnetism and diamagnetism on susceptibility-weighted imaging signals (Eskreis-Winkler et al., 2017). QSM has been used in several studies to quantify iron or copper levels in patients with WD (Fritzsch et al., 2014; Dusek et al., 2017, 2018; Saracoglu et al., 2018). The current study used QSM to quantify changes in magnetic susceptibility in TX mouse brain samples. Our data showed that the susceptibilities of all analyzed regions were diminished across different periods of treatment. Increased magnetic susceptibility relative to the reference region generally indicates the presence of paramagnetic minerals such as iron and copper (II) compounds, in the brain. Our correlation analysis showed that the changes in the magnetic susceptibilities of the TX mouse brain samples reflected changes in the copper concentrations. Thus, possibly the observed increases in susceptibilities were caused by elevated concentrations of a paramagnetic copper (II) compound. Analysis of the T2 maps revealed findings unlike those from the QSM data. Differences between the T2 values in the quantitative T2 maps could not be shown in our study. The MRI signal changes can reflect reversible myelin sheath injury, cytotoxic oedema, cavernous degeneration and necrosis caused by copper poisoning. In the early stage of WD, diffusion of corpus striatum was limited. In our study, we found that when T2W images were normal, the magnetic susceptibility of cortex, corpus striatum, hippocampus and cerebellum increased significantly. This may be because at this time in the early stage of copper deposition, it has not caused obvious organic damage. Perhaps after statistically increasing the accumulation of copper in the brain, differences between the T2 values will appear.

Our study was also subject to limitations. First, since we exclusively focused on TX mice, the study group was relatively small, and the conclusions drawn from this small population may be limited. Therefore, further studies that include larger samples of animals and patients with WD are needed to validate the results of this study. Second, we only focused on QSM of the cortex, striatum, hippocampus, amygdaloid nucleus, and cerebellum; thus, we may have overlooked potential findings in the brain stem, which is related to the neural circuits affected by WD.

## Conclusion

Our results provide evidence that PCA treatment and copper accumulation in the brain is associated with the worsening of neurological symptoms in TX mice. Additionally, our findings suggest that there is a potential connection between copper depositions and the magnetic susceptibilities of the cortex, corpus striatum, hippocampus, and cerebellum of TX mice. Finally, our study demonstrates that QSM and ICP-MS can be used to study the spatial distribution of copper deposits in the brains of WD mice. Overall, our results confirmed that copper is significantly redistributed following PCA treatment, and this finding may improve our understanding of the mechanisms underlying the PCA-induced worsening of symptoms in patients with WD and also help develop future treatment strategies for WD.

## Data Availability Statement

All datasets generated for this study are included in the article.

## Ethics Statement

The animal study was reviewed and approved by Anhui University of Chinese Medicine Animal Ethics Committees.

## Author Contributions

YH and JD: designed and performed experiments, analyzed data and wrote the article. YW and HY: acquisition, analysis, and interpretation of data, agreement to be accountable for all aspects of the work in ensuring that questions related to the accuracy or integrity of any part of the work are appropriately investigated and resolved. CX: performed experiments and analyzed data. RR, SS, and GL: acquisition, analysis, and interpretation of data; drafting and revising the manuscript. NC: interpretation of data, revising the manuscript. YH and KZ: designed experiments, analyzed data and final approval of the submission.

## Conflict of Interest

The authors declare that the research was conducted in the absence of any commercial or financial relationships that could be construed as a potential conflict of interest.
